# Local versus offshore production of ready‐to‐use therapeutic foods and small quantity lipid‐based nutrient supplements

**DOI:** 10.1111/mcn.12376

**Published:** 2016-11-08

**Authors:** Joel Segrè, Grace Liu, Jan Komrska

**Affiliations:** ^1^ Independent Consultant Oakland CA USA; ^2^ Independent Consultant Cambridge MA; ^3^ Former contracts manager, UNICEF Copenhagen Denmark

**Keywords:** lipid‐based nutrient supplement (LNS), local production, manufacturing, offshore production, ready‐to‐use therapeutic foods (RUTF)

## Abstract

Manufacturers on four continents currently produce ready‐to‐use therapeutic foods (RUTF). Some produce locally, near their intended users, while others produce offshore and ship their product long distances. Small quantity lipid‐based nutrient supplements (SQ‐LNS) such as Nutriset's Enov'Nutributter are not yet in widespread production. There has been speculation whether RUTF and SQ‐LNS should be produced primarily offshore, locally, or both. We analyzed The United Nations Children's Fund (UNICEF) Supply Division data, reviewed published literature, and interviewed local manufacturers to identify key benefits and challenges to local versus offshore manufacture of RUTF. Both prices and estimated costs for locally produced product have consistently been higher than offshore prices. Local manufacture faces challenges in taxation on imported ingredients, low factory utilization, high interest rates, long cash conversion cycle, and less convenient access to quality testing labs. Benefits to local economies are not likely to be significant. Although offshore manufacturers offer RUTF at lower cost, local production is getting closer to cost parity for RUTF. UNICEF, which buys the majority of RUTF globally, continues to support local production, and efforts are underway to narrow the cost gap further. Expansion of RUTF producers into the production of other ready‐to‐use foods, including SQ‐LNS in order to reach a larger market and achieve a more sustainable scale, may further close the cost and price gap. Local production of both RUTF and SQ‐LNS could be encouraged by a favorable tax environment, assistance in lending, consistent forecasts from buyers, investment in reliable input supply chains, and local laboratory testing.

## 1 BACKGROUND

Worldwide, an estimated 17 million children under age 5 years suffer from severe acute malnutrition, with an additional 34 million suffering from moderate acute malnutrition and 161 million in total suffering from chronic malnutrition (UNICEF, WHO, The World Bank, 2014). These prevalence estimates are likely lower than yearly incidence for acute malnutrition. Malnutrition plays a role in one‐third of deaths under‐5 years each year (WHO, 2013).

Since 2007, the World Health Orgamozation has recommended the use of ready‐to‐use therapeutic foods (RUTF) for treating severe acute malnutrition in children with appetite and without complications (WHO, WFP, UNSSCN & UNICEF, 2007). RUTF are lipid‐based foods with low water content, providing a long shelf life and safe consumption out of the package, thereby facilitating effective community‐based management. In recent years, ready‐to‐use foods (RUF) have expanded to include supplementary food in addition to therapeutic food. Ready‐to‐use supplementary food (RUSF) most commonly refers to large‐quantity lipid‐based nutrient supplements for treatment of moderate acute malnutrition, a convention we will follow here. Other supplementary food products include medium‐quantity lipid‐based nutrient supplement to treat chronic malnutrition and prevent acute malnutrition, and small‐quantity lipid‐based nutrient supplement (SQ‐LNS) designed to prevent chronic malnutrition and stunting. Prevention of malnutrition is especially critical during the first 1,000 days from conception to 2 years of age, the period during which nearly all stunting occurs, impacting health throughout life (Black et al., 2013) Though RUSF use has increased, SQ‐LNS use remains limited.

Roughly half the global supply of RUTF is currently produced by Nutriset, the French innovator of Plumpy'Nut®, the first commercially available RUTF product, developed in 1996 (Nutriset, [Link]). Since 2005, Nutriset has shared its patented formulation with other manufacturers through its PlumpyField Network, aimed at increasing “nutritional autonomy” for developing countries. The PlumpyField Network now comprises Nutriset, seven partners in low‐income countries, and a US‐based nonprofit organization (PlumpyField, [Link]). The United Nations Children's Fund (UNICEF) is the largest purchaser of RUTF and further supports local production, with a goal of reaching 50% sourcing from programmatic countries by 2016 (UNICEF Supply Division, [Link]). RUTF is frequently used in emergency situations as well as in community‐based management of acute malnutrition programs (Collins, 2010).

The shift from offshore to local production reflects a recognition of the importance of food security for vulnerable populations and their children, as well as perceived advantages including long‐term cost reduction, increased availability, economic benefits to farmers and manufacturers, and customization to locally available ingredients, preferred tastes, and distribution channels. However, local manufacturers also encounter specific challenges to producing safe and cost‐competitive products. Neither the suggested economic benefits nor the challenges of local production have been well‐explored in the literature.

This paper aims to aggregate available data on RUF production, to examine quantitatively and qualitatively the benefits and challenges to local RUF production, and to identify potential ways to create a more favorable local manufacturing environment. Analysis focuses on RUTF procurement and production where data is available, namely the ~80% of the RUTF market procured by UNICEF, and the ~70% of global market produced by Nutriset and the PlumpyField network (Van Pel, Newton, & Twiss, 2015).

**Key messages**

- Local production of RUF remains more costly than offshore production, yet local production is necessary to ensure that countries can prevent and treat malnutrition within their borders, and move toward self‐sufficiency.
- Donors should continue to procure locally, and be transparent that the added costs are necessary to strengthen a fledgling industry that could one day serve the majority of RUTF needs, and expand production to locally customized SQ‐LNS products.

## 2 METHODS

We analyzed UNICEF Supply Division data on suppliers, pricing, freight, taxation, and production capacity. Some data on pricing and procurement are publicly available (UNICEF, 2014; UNICEF, 2015a).

We searched PubMed for published articles on RUTF pricing, costs, and benefits and challenges of offshore versus local production, and additionally reviewed grey literature from community‐based management of acute malnutrition forum (CMAM Forum, 2015) iLiNS Project (iLiNS 2015), and UNICEF Supply Division (UNICEF, 2015b).

We additionally conducted site visits and interviews with local and offshore manufacturers to identify other benefits and challenges to local production.

For this analysis, a producer is described as “local” if it is based in a UNICEF programmatic country with high levels of undernutrition. All other producers are described as “offshore.”

## 3 RESULTS

As of July 2015, UNICEF reported a supplier base of 15 manufacturers of RUTF, including nine local manufacturers in program countries. Of the nine local manufacturers, seven had local prices, two provided product locally and for export, and two exclusively exported product.

### 3.1 Local versus offshore pricing

**Figure 1:**
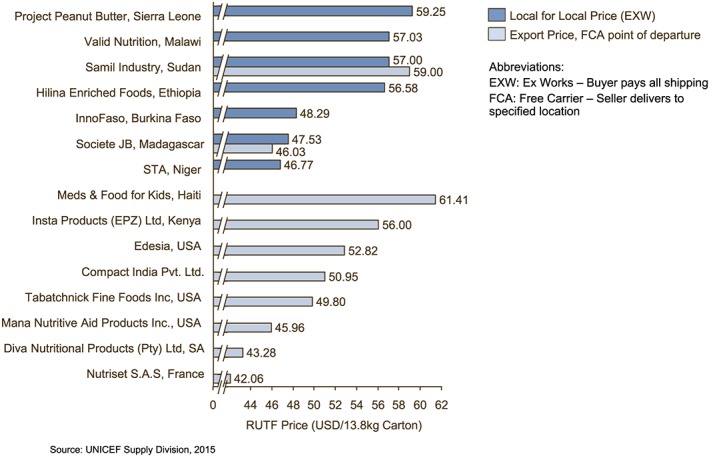
The United Nations Children's Fund pricing for ready‐to‐use therapeutic foods (RUTF), July 2015

**Figure 2:**
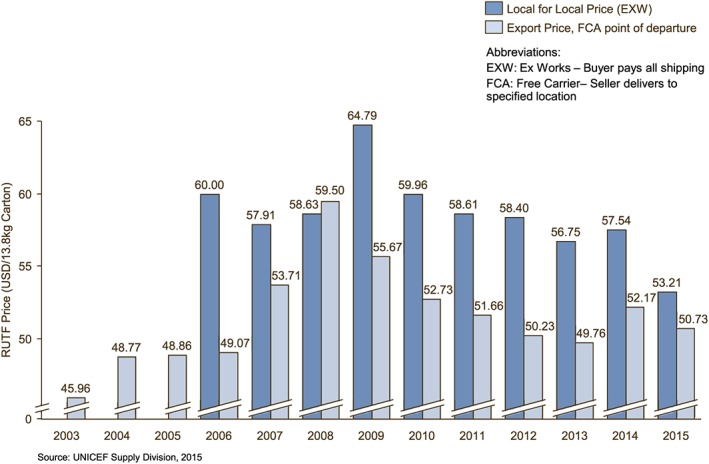
The United Nations Children's Fund local and export pricing for ready‐to‐use therapeutic foods (RUTF), 2003–2015

Figure 1 shows the 2015 prices per carton (150 sachets of 92 g each) for all 15 manufacturers, including the two that produced RUTF for both export and local use. The average price from offshore manufacturers was 47.48 USD per carton versus the average local for local price of $53.21. Figure 2 shows the evolution of offshore and local‐for‐local prices since 2003 and the narrowing price gap. All prices in Figures 1 and 2 are exclusive of international transport.

### 3.2 Transportation

**Figure 3:**
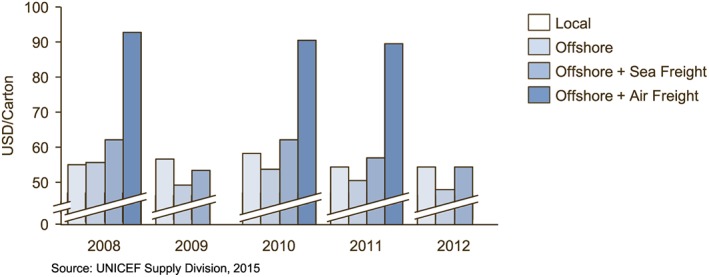
Offshore versus local production in a tax free environment of Ethiopia

Figure 3 shows a cost comparison of RUTF manufactured in Ethiopia by Hilina Enriched Foods Processing Center against RUTF manufactured offshore by Nutriset, with and without cost of freight. Both the offshore product and imported ingredients for the local producer were exempt from import duties. In this relatively rare tax free environment, local production is near parity with offshore production plus sea freight.

### 3.3 Taxation

**Figure 4:**
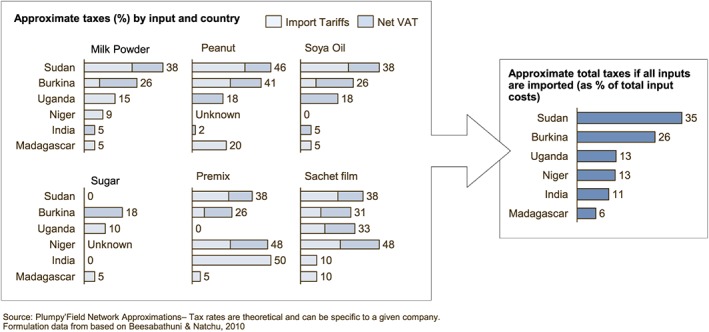
Estimated tax burden for 2012 in selected countries. VAT, value added tax

The United Nations Children's Fund imports RUTF into high‐need countries free of both import duties and value added tax (VAT). Local producers in some countries are subject to import duties for specific inputs they cannot source locally, and VAT on all inputs, regardless of origin. Figure 4 summarizes estimated 2012 tax rates on standard inputs in six countries with local production of RUTF. If all inputs are imported, tax rates constitute 6–35% of total input cost, in some cases making offshore RUTFs less expensive, after shipping, than local production.

### 3.4 Production capacity and factory utilization

**Figure 5:**
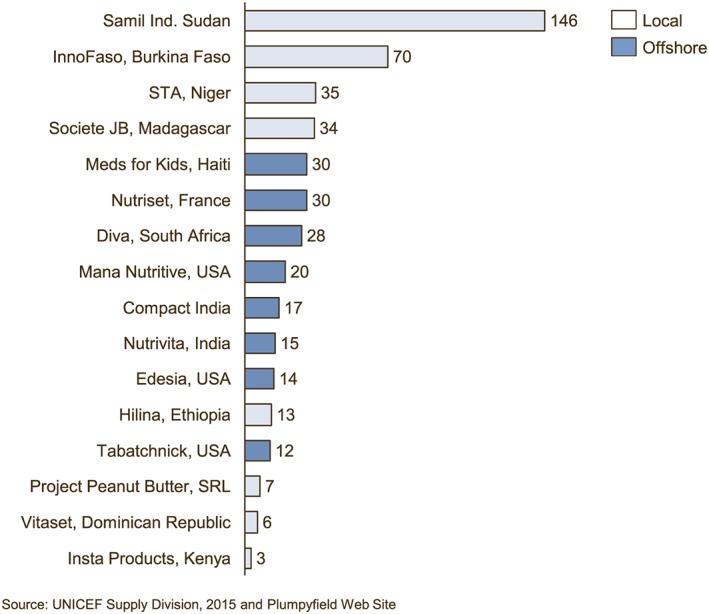
2014 RUTF sales to UNICEF as a percentage of stated capacity

Table 1 shows the stated production capacity and utilization by UNICEF in 2014 across a base of 20 suppliers (including the 17 with pricing in 2015). Offshore manufacturers had three times the total production capacity of local manufacturers, providing 75% of total production capacity, 72% of production capacity allocated to UNICEF, and 76% of RUTF purchased by UNICEF. UNICEF orders in the first half of 2015 totalled 14,714 MT, with 74% from offshore manufacturers. Figure 5 shows the UNICEF procurement as a percentage of stated capacity for individual manufacturers in 2014.

**Table 1:** RUTF production capacity and utilization for UNICEF suppliers in 2014

Manufacturer location	Production capacity		UNICEF procurement			
	Stated total (MT)	Percentage allocated to UNICEF	Amount (MT)	Percentage of allocated capacity	Percentage of total capacity	Percentage of total procurement
Local	34,673	68	7,398	32	21	24
Offshore	98,400	58	23,042	40	23	76
All	131,873	60	33,566	42	25	100

Source: UNICEF Supply Division, 2015. RUTF = ready‐to‐use therapeutic foods; UNICEF = The United Nations Children's Fund.

### 3.5 Cost of debt

Local producers stated in interviews that working capital is in short supply for most local RUTF producers. Most must borrow working capital to operate. Cost of debt varies due to differences in interest rates and in the duration of the loan required. Banks in low‐income countries typically charge higher interest rates compared with banks in high‐income countries. Credit from vendors is also more readily available to European and American producers, which benefit from a legal infrastructure offering security to creditors in case of default. As a result, local producers are more likely to be required to pay for inputs in advance. In addition, imported inputs take longer to reach local producers, which most also ship product offshore for laboratory analysis and quality control.

**Figure 6:**
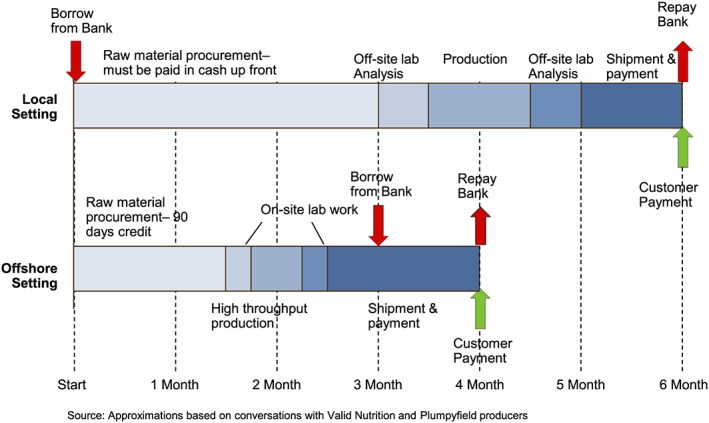
Cash conversion cycles in offshore versus local contexts

Interviews with Nutriset and Valid Nutrition provided information on typical cash conversion cycles of an offshore and a local producer, respectively. Figure 6 compares the cash conversion cycles. The total difference in production time is 2 months. In this illustrative case, the local producer borrows for a full 6 months, compared to 1 month for the offshore producer.

We estimate the total cost of debt using the illustrative cash conversion cycles with 2014 lending rates given by the World Bank. Lending interest rates for five countries with local production and three countries with offshore production (all UNICEF supplier countries with reported lending interest rates) and resulting costs of debt are shown in Table 2. In the most extreme case, the estimated cost of debt for a manufacturer in Madagascar is two orders of magnitude larger than the cost of debt for a manufacturer in the United States.

**Table 2:** Estimated cost of debt in countries with local and offshore production

	Lending interest rate (%)	Estimated cost of debt per 100,000 USD of principal ($)
Program countries		
Haiti	10.8	5,400
Tanzania	16.2	8,100
Kenya	16.5	8,250
Malawi	44.3	22,150
Madagascar	60.0	30,000
Non‐program countries		
United States	3.3	275
South Africa	9.1	758
India	10.3	858

Assume 2014 World Bank lending interest rate and loan duration of 6 months for local and 1 month for offshore manufacturers.

### 3.6 Quality/laboratory analysis

Most local manufacturers send samples overseas to be tested for microbial contaminants and aflatoxin from peanuts. To address this barrier in Ethiopia, the local manufacturer Hilina Enriched Foods was a partner in the creation of a new laboratory for local analysis, Bless Agri Food Laboratory Services.

### 3.7 Procurement from local manufacturers

**Figure 7:**
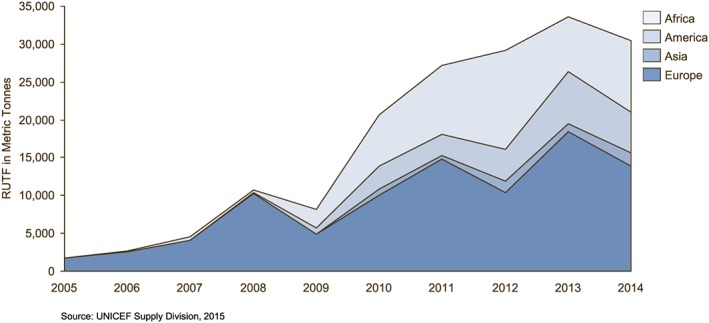
Local and offshore ready‐to‐use therapeutic food procurement from The United Nations Children's Fund

The United Nations Children's Fund Supply aims to increase procurement from local manufacturers to 50% by 2016. In 2012, UNICEF procured a high of 45% of RUTF from manufacturers based in Africa, but this share decreased to 31% in 2014 (Figure 7) and was at 28% in mid‐2015.

### 3.8 Local economic benefit

For the three local producers for whom UNICEF had employment data, full‐time employees totalled 91. PlumpyField estimated over 500 new jobs have been created through the entire network since 2005. This estimate does not include indirect creation of jobs in laboratories, agricultural supply chains, transportation, and other related industries.

## 4 DISCUSSION

### 4.1 Ready‐to‐use therapeutic foods

Pricing data show that year over year, locally produced RUTF has been more expensive than offshore production. The disparity in average prices before shipping costs has decreased since to less than 5% in 2015, with the advantage of lower shipping costs for local product. Still, local production does not yet meet local global demand.

**Taxation** raises RUTF costs for local producers where taxable inputs are imported due to an absence of local availability (e.g., milk powder in markets where evaporators are not present) or quality standards (e.g., peanuts in peanut producing countries which suffer from aflatoxin contamination). Tax exemptions from both import duties and VAT would encourage local production to be more cost competitive.

**Low factory utilization** may also contribute to production cost. Factories with minimal idle capacity have lower fixed costs per unit production, resulting in lower prices. Keeping idle capacity low can be a challenge for RUTF producers, whose demand can spike or evaporate in response to emergencies and variations in donor funding. This has an important and counterintuitive implication for competition within the RUTF market. Introduction of large new producers runs the risk of increasing the price for RUTF across the industry because the market is still relatively small. Increases in capacity without commensurate increases in demand can mean that all manufacturers have greater idle capacity across the industry, and potentially, higher cost per unit. In 2009–2011, the largest three funding sources, comprising 49% of total funding (UNICEF Supply Division, 2015), were donors who focus on emergencies. High‐volume offshore producers may have a utilization advantage in aggregating these demand spikes across multiple products, but capacity utilization among both offshore and local suppliers to UNICEF remains low. This is true despite UNICEF Supply goals of increased procurement from program countries.

**Cost of debt** and the resulting working capital constraints can handicap local producers. This also suggests why most producers (Valid Nutrition, 2012) do not maintain a significant buffer stock. They simply cannot afford to have scarce working capital tied up in product sitting on warehouse shelves, particularly in view of uncertain local demand and shelf life constraints.

**Quality assurance** is a critical concern of all producers but poses special challenges in local production environments.

#### 4.1.1 Laboratory analysis

Costs for laboratory analysis of inputs and finished products have been rising in recent years for RUTF producers and have proven especially costly for local manufacturers who must send samples overseas due to lack of certified local laboratories (Van Pel, et al., 2015). In order to address this issue, Hilina Enriched Foods and Onyx Développement invested several million euros in the creation of Bless Agri Food Laboratory Services in Ethiopia (Duclercq, 2014; PlumpyField, 2013). In addition to providing local services to Hilina since 2013, Bless Agri Food is expected to benefit RUF manufacturers across East Africa as well as provide services to unrelated food producers in the region. The laboratory also aims to increase awareness of food safety issues including aflatoxin in peanuts (Agonifer, [Link]) and aid in sourcing safe local peanuts.

#### 4.1.2 Peanuts

Peanuts are carefully regulated on their concentration of aflatoxin, which is produced by fungal contamination of peanuts before they reach manufacturers for processing (Manary, 2006). Stringent aflatoxin limits frequently require local RUF producers to import peanuts even when peanuts are domestically available (Van Pel, et al., 2015). Unsafe peanuts have even forced production to shut down (Duclercq, 2014).

To address this problem, some local producers have invested in the improvement of local agricultural practices in order to improve their supply chain (Ortiz Nunez, 2010). Improving local peanut crops increases near‐term cost and complexity but has potential long‐term benefits. Agreements between RUF manufacturers and farmers may help to provide stable markets for peanuts, facilitate third‐party investment in improved peanut processing facilities, and create an entry point for a peanut processing industry with larger markets. While some have argued that investment in local crops for RUF production would improve food security (rather than undermining local markets and increasing dependence on imports; Collins, 2010), others have argued that the safety standards for RUF production are so stringent that these agricultural outputs are effectively new products, priced out of local markets.

#### 4.1.3 Microbial contaminants

Unsafe processing within the RUF production facility may also lead to microbial contamination of the final product with Salmonella and Enterobacteriaceae (FAO & WHO, 2013). In 2012, *Cronobacter sakazakii* was detected in RUTF samples, leading to increased testing and production shortages among local manufacturers (Duclerq 2014), as seen in the drop‐off in procurement from UNICEF program countries in 2012–2013 (Figure 7). Concerns over *C. sakazakii* also led to a broader discussion of RUF safety, with the Codex Alimentarius Commission working to develop an official guideline for RUTF (FAO & WHO, 2015). To minimize microbial contaminants like Enterobacteriaceae and Salmonella, a thermo‐processing kill step is recommended but not required.

#### 4.1.4 Other potential benefits of local production

##### 4.1.4.1 Local economic benefit

Proponents of local manufacture have argued that local manufacturers generate local employment and purchase local inputs (Collins, 2010; FAO & WHO, 2015). While the number of jobs may not be enough to have a substantial impact on the local economy, there is an argument that these are relatively high quality jobs, offering good stability, wages, and improved skills that can create ripple effects in local communities, especially as local manufacturers bring new skills to a region, and employees may carry those skills into future jobs. Reliable data on these indirect benefits are not readily available.

##### 4.1.4.2 Product customization to local inputs, tastes, and distribution channels

In the case of RUTF, UNICEF buys an estimated 80% of total global production using a specific set of tender specifications based on tested therapeutic regimens for treating severe acute malnutrition (WHO et al., 2007), which can restrict country‐level customization. Moreover, RUTF distribution is often free of charge, and end users have little input regarding product preferences. However, research and development on improved RUTF is active and purchasers are considering new formulations. Linear programming models identify alternative formulations, optimized for cost and locally available or preferred ingredients while meeting the same nutritional requirements (Dibari, Diop, Collins, & Seal, 2012).

Valid Nutrition and Hilina Foods, among others, have been working on alternative formulations of lipid‐based products that reduce dependency on expensive imported inputs in favor of lower cost, locally available inputs like chickpeas. Vietnam publicly procures and runs programs using a locally developed RUTF formulation based on moon cake (Fleet, 2015) a traditional baked good containing bean paste.

Manufacturers with local roots may be better positioned to identify these opportunities to simultaneously cut cost and improve local economic impact. However, R&D capacity is still emerging among these local manufacturers, and in the Hilina and Valid cases above, they rely on foreign technical assistance to refine and validate their products.

##### 4.1.4.3 Local government engagement

Critically, local production can help national governments tighter integration with programs addressing malnutrition. Local ownership could improve coordination across manufacturing, agricultural supply chain, distribution channels, health systems, community‐based management, and local needs and tastes. When well executed, this increases the capacity of governments to deliver on commitments to adequate child nutrition.

### 4.2 Small quantity lipid‐based nutrient supplements

Expanding production from RUTF to RUSF and SQ‐LNS could potentially assist local manufacturers in achieving sustainable scale. All RUF products require similar raw materials and safety standards. RUSF and SQ‐LNS are composed of the same ingredients as RUTF in differing proportions, and RUSF is subject to the same microbial and aflatoxin limits as RUTF (FAO & WHO, 2013). Expansion to RUSF and SQ‐LNS production could therefore be expected to require limited further investment, while increased scale would improve pricing and support investment in safe food processing and local or regional laboratory testing.

Small quantity lipid‐based nutrient supplement is potentially a much larger market, as preventive needs are greater than therapeutic ones. With need likely outstripping philanthropic potential, there is interest in exploring commercial sales. A study of Ethiopian households found some willingness to pay for a week's supply of SQ‐LNS among nearly all surveyed urban households, with a quarter willing to pay a projected unsubsidized commercial price (Segrè et al., 2012).

However, SQ‐LNS as a preventive rather than therapeutic product for acute malnutrition brings a new set of challenges as well as potential benefits.

#### 4.2.1 Taxation

As a non‐emergency (and potentially retail) product, offshore producers of SQ‐LNS are likely to be subject to taxation. This means that taxation on SQ‐LNS might not drive cost differences between local and offshore producers to the extent observed in the RUTF market.

Both finished products from overseas and inputs used for local manufacture would likely be taxed. As the SQ‐LNS made its way to retail sale, VAT would likely be applied at the same rate regardless of offshore or local origin.

#### 4.2.2 Production capacity and factory utilization

Factory utilization is likely to be more predictable for SQ‐LNS than it is for RUTF. Rather than responding to emergencies or relying on cycles of donor funding, SQ‐LNS as a preventive product could be expected to behave more like commercially available complementary foods or other nutritious products targeting women of reproductive age, pregnant mothers, and children. If retail sales become successful, SQ‐LNS would have steadier, more predictable demand, enabling much better matches between factory capacity and factory utilization. Factories producing SQ‐LNS and RUTF might also be better able to manage spikes in RUTF demand by adjusting their baseline volume of SQ‐LNS.

#### 4.2.3 Local economic benefits

**Figure 8:**
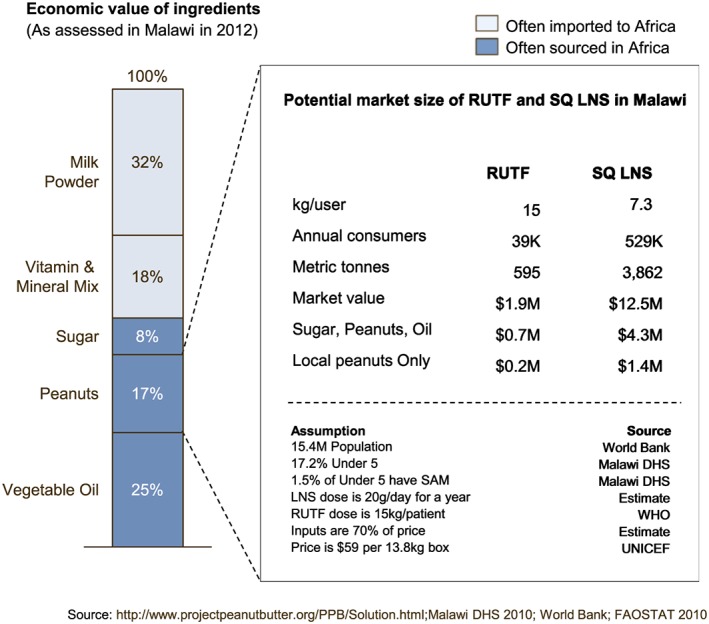
Estimate of economic value of local ready‐to‐use therapeutic foods (RUTF) and lipid‐based nutrient supplements production. WHO, World Health Organization; SQ‐LNS, small quantity lipid‐based nutrient supplements.

In terms of purchase of local inputs, the volume of RUTF and SQ‐LNS produced is unlikely to have a substantial impact on agriculture at the country level. In Malawi, for example, where there have been two separate efforts to produce RUTF, ingredients are often imported, and the quantities of peanuts, oil, and sugar needed for RUTF constitute a negligible portion of the country's total agricultural output (Manary, 2006). Only 25–50% of the economic value of the inputs for today's RUTF or SQ‐LNS formulations is likely to be sourced within the country (Figure 8). According to the Statistics Division of the United Nations Food and Agriculture Organization, Malawi produced about $176 million worth of peanuts across the country in 2010. Assuming that local producers could serve every child aged 6–18 months with SQ‐LNS for one year and every child with severe acute malnutrition with RUTF, the aggregate peanut demand for SQ‐LNS and RUTF combined would be around $1.6 M USD, or 0.8% of annual peanut production. Such a volume might not greatly impact the growth of the local peanut industry but could prompt some producers to abide by improved general safety standards, especially for aflatoxin. Expanding SQ‐LNS consumption to include all women of reproductive age (however, implausible) would boost the volume to 6% of national production.

Additional production of SQ‐LNS would likely require additional employment, but production volume and employment levels are not well correlated, as equipment choices and the level of automation varies from site to site.

#### 4.2.4 Quality

All RUF are low‐moisture foods not requiring reconstitution with water, thus are an unlikely medium for bacterial growth. Bacteria may be preserved in a metabolically dormant state, producing concerns for immune‐compromised, malnourished children. From a production point of view, no RUF is significantly more prone to quality problems than another. The difference is in the usage case. The smaller quantity and healthier consumers of SQ‐LNS yield a lower safety risk associated with the product relative to RUTF. As standards develop, safety requirements for SQ‐LNS are therefore unlikely to be more stringent than for RUTF and RUSF.

#### 4.2.5 Customization

For SQ‐LNS, local manufacturers may offer additional insights into potential customization. As a preventive product, SQ‐LNS is designed with a much larger consumer group in mind. A successful, large‐scale SQ‐LNS program is likely to engage private sector retail outlets for distribution in populations where families are able to buy SQ‐LNS along with other foods and food supplements. In this scenario, SQ‐LNS would be in competition with other products for a family's budget and could benefit from local customization to meet local expectations around branding, packaging, price, and taste. As with RUTF, however, local insights into consumer demand will need to be matched with capacity to conduct R&D and produce novel, quality products.

Given the similarity of the ingredients and manufacturing processes, the strongest candidates for local production of SQ‐LNS will likely be current producers of RUTF. These organizations, however, may not have the skillset to develop successful retail products, as they may be more accustomed to institutional buyers like UNICEF. In this case, local and offshore producers may benefit from partnerships with local food producers and distributors who might understand local distribution channels, brands, and tastes better.

The final area where local customization could help is in demand generation. As a relatively unknown product, SQ‐LNS could face significant challenges in getting families familiar with the product category and benefits. This presents business risk to offshore and local producers alike, and each will have advantages in mitigating that risk. On the one hand, local producers may know what kind of promotion can reach consumers best. On the other hand, offshore producers may be more likely to have the budget to invest in demand generation activities. In regions where there may be resistance to advertising of products for children under 2 years of age, again, a combination of local political understanding from local manufacturers combined with global experience of offshore producers could be useful. In all cases, demand generation for SQ‐LNS distributed through retail channels remains among the greatest risks to all potential investors in SQ‐LNS production, regardless of their location.

### 4.3 Creating a favorable environment for local production of RUTF and SQ‐LNS

Despite the challenges of local manufacturing, we believe that the future of RUF manufacturing can and should ultimately rest with local producers providing food security to their own populations. Nutriset's PlumpyField network is accelerating the transition from offshore to local production, helping member producers reduce their costs, improve their quality, and initiate sales. To reduce costs, PlumpyField pools procurement across multiple producers to take advantage of volume discounts on inputs that must be imported to most low income countries, like vitamin mix. The network also standardizes production equipment to reduce technical risks, streamline procedures, and ease the burden of maintenance and repairs. PlumpyField also improves member access to working capital, effectively lending at a rate otherwise unattainable in the local context. Nutriset's technical expertise is helpful in assisting PlumpyField members to develop formulations that reduce cost, serve different nutritional needs, or meet local tastes. The network offers support to member quality management systems, improving quality audit results and reducing downtime. Finally, STA and Nutriset have invested in the only known commercial launch of SQ‐LNS, Grandibien in Niger. These types of market pilots help to demonstrate the conditions required for consumer demand, reducing risk for local manufacturers, distributors, and retailers.

The proportion of RUTF sourced locally will also likely continue to rise. In some countries, the opportunity to procure from local manufacturers can be instrumental in getting national governments to consider allocation of funding for RUTF purchase.

Findings here are limited by the data available from UNICEF and difficulty in obtaining data from individual companies. The RUTF market has also seen rapid change, while RUSF and SQ‐LNS are very new products. However, it is clear that several actions could be taken to accelerate the shift to local production of RUF products:

First, governments of producing countries could significantly improve the competitiveness of local industry by waiving duties and VAT on inputs for RUTF. Second, high‐volume buyers of RUTF and SQ‐LNS products could consider measures to shorten the cash conversion cycle for local manufacturers, alleviating their cost of debt and working capital shortage. Third, greater effort on the part of buyers to forecast and smooth their order volumes would help manufacturers around the world to operate more efficiently, better matching production capacity with order size. Fourth, investment in local quality testing facilities would reduce both the length of the cash conversion cycle and the cost of quality testing. Finally, regardless of origin, SQ‐LNS will face significant challenges with demand creation. Local regulation of product promotion targeting the first 1,000 days of life, in particular, will be a limiting factor on promotion of these products to their target users, and therefore product sales.

As in any new industry, the first investments made in local manufacturing of RUTF and SQ‐LNS were risky, but these risks are decreasing with time. While investments in local production are not changing their host countries overnight, they are sowing seeds for local growth. Over time, if local producers flourish, they will strengthen their local economies while improving product, price, and availability for all.

## SOURCE OF FUNDING

This publication is based on research funded in part by the Bill & Melinda Gates Foundation. The findings and conclusions contained within are those of the authors and do not necessarily reflect positions or policies of the Bill & Melinda Gates Foundation.

## CONFLICTS OF INTEREST

The authors declare that they have no conflicts of interest.

## CONTRIBUTIONS

JS designed the study, collected and analyzed data from primary and secondary sources and wrote the manuscript. GL collected and analyzed data from secondary sources and wrote the manuscript. JK collected and analyzed data from primary sources and reviewed the manuscript. All approved the manuscript for submission.
